# Modeling road traffic fatalities in Iran’s six most populous provinces, 2015–2016

**DOI:** 10.1186/s12889-022-14678-5

**Published:** 2022-11-30

**Authors:** Fatemeh Jahanjoo, Homayoun Sadeghi-Bazargani, Mohammad Asghari-Jafarabadi

**Affiliations:** 1 Road Traffic Injury Research Center, Tabriz University of Medical Sciences, Tabriz, 5167846311 East Azerbaijan Islamic Republic of Iran; 2 Injury Epidemiology and Prevention Research Group, Turku Brain Injury Center, Turku University Hospital and the University of Turku, Turku, Finland; 3 Cabrini Research, Cabrini Health, Melbourne, VIC 3144 Australia; 4 School of Public Health and Preventative Medicine, Faculty of Medicine, Nursing and Health Sciences, Monash University, Melbourne, VIC 3800 Australia

**Keywords:** Road traffic injury, Statistical modelling, Driving behaviour, Road factors, Iran

## Abstract

**Background:**

Prevention of road traffic injuries (RTIs) as a critical public health issue requires coordinated efforts. We aimed to model influential factors related to traffic safety.

**Methods:**

In this cross-sectional study, the information from 384,614 observations recorded in Integrated Road Traffic Injury Registry System (IRTIRS) in a one-year period (March 2015—March 2016) was analyzed. All registered crashes from Tehran, Isfan, Fras, Razavi Khorasan, Khuzestan, and East Azerbaijan provinces, the six most populated provinces in Iran, were included in this study. The variables significantly associated with road traffic fatality in the uni-variate analysis were included in the multiple logistic regression.

**Results:**

According to the multiple logistic regression, thirty-two out of seventy-one different variables were identified to be significantly associated with road traffic fatality. The results showed that the crash scene significantly related factors were passenger presence(OR = 4.95, 95%CI = (4.54–5.40)), pedestrians presence(OR = 2.60, 95%CI = (1.75–3.86)), night-time crashes (OR = 1.64, 95%CI = (1.52–1.76)), rainy weather (OR = 1.32, 95%CI = (1.06–1.64)), no intersection control (OR = 1.40, 95%CI = (1.29–1.51)), double solid line(OR = 2.21, 95%CI = (1.31–3.74)), asphalt roads(OR = 1.95, 95%CI = (1.39–2.73)), nonresidential areas(OR = 2.15, 95%CI = (1.93–2.40)), vulnerable-user presence(OR = 1.70, 95%CI = (1.50–1.92)), human factor (OR = 1.13, 95%CI = (1.03–1.23)), multiple first causes (OR = 2.81, 95%CI = (2.04–3.87)), fatigue as prior cause(OR = 1.48, 95%CI = (1.27–1.72)), irregulation as direct cause(OR = 1.35, 95%CI = (1.20–1.51)), head-on collision(OR = 3.35, 95%CI = (2.85–3.93)), tourist destination(OR = 1.95, 95%CI = (1.69–2.24)), suburban areas(OR = 3.26, 95%CI = (2.65–4.01)), expressway(OR = 1.84, 95%CI = (1.59–2.13)), unpaved shoulders(OR = 1.84, 95%CI = (1.63–2.07)), unseparated roads (OR = 1.40, 95%CI = (1.26–1.56)), multiple road defects(OR = 2.00, 95%CI = (1.67–2.39)). In addition, the vehicle-connected factors were heavy vehicle (OR = 1.40, 95%CI = (1.26–1.56)), dark color (OR = 1.26, 95%CI = (1.17–1.35)), old vehicle(OR = 1.46, 95%CI = (1.27–1.67)), not personal-regional plaques(OR = 2.73, 95%CI = (2.42–3.08)), illegal maneuver(OR = 3.84, 95%CI = (2.72–5.43)). And, driver related factors were non-academic education (OR = 1.58, 95%CI = (1.33–1.88)), low income(OR = 2.48, 95%CI = (1.95–3.15)), old age (OR = 1.67, 95%CI = (1.44–1.94)), unlicensed driving(OR = 3.93, 95%CI = (2.51–6.15)), not-wearing seat belt (OR = 1.55, 95%CI = (1.44–1.67)), unconsciousness (OR = 1.67, 95%CI = (1.44–1.94)), driver misconduct(OR = 2.51, 95%CI = (2.29–2.76)).

**Conclusion:**

This study reveals that driving behavior, infrastructure design, and geometric road factors must be considered to avoid fatal crashes. Our results found that the above-mentioned factors had higher odds of a deadly outcome than their counterparts. Generally, addressing risk factors and considering the odds ratios would be beneficial for policy makers and road safety stakeholders to provide support for compulsory interventions to reduce the severity of RTIs.

## Introduction

Iran has a serious problem with high traffic levels above average due to several factors, including transportation strategies and sociocultural and economic features. Regarding to the world Health Organization (WHO) data published in 2020, the number of deaths due to road traffic injuries (RTIs) exceeded deaths from heart diseases in Iran [1]. It has been also reported that traffic accidents caused approximately 100,000 fatalities and more than 2 million serious injuries over an 8-year period from 2013 to 2020 [2].

So far, global initiatives have sought to understand better and address the underlying mechanisms of road safety, many of which are aligned with the worldwide program of the Decade of Action for Road Safety 2011–2020 prepared by the United Nations Road Safety collaboration (UNRSC) [3]. However, despite the increase in road injuries in Iran, the main reasons for such an important issue have not been appropriately identified. Based on the reports by the head of traffic information and control center of Iran traffic police, driver fault was the primary factor in traffic accidents [4]. Although driver fault features top the list of causes in Iran, other elements cannot be neglected. There are more causes of traffic accidents, such as environmental, road-related, road-user, vehicle, and driver-related factors. Valuable existing studies have identified only part of the risk factors for RTIs, and there is no comprehensive study in this field yet. For example, Lankarani et al. (2014) aimed to address environmental factors in road traffic crashes. They used data from a cross-sectional study of the traffic police department between March 2010 and December 2010. The results indicated that day time, dusty weather, oily road surfaces, ominous traffic signs, road narrowing, and downhill roads were highly correlated with road crash-related deaths [5]. A study by Sherafati et al. (2017) showed that crash severity and length of admission time were the leading causes of inequity in fatality rates between urban and rural regions [6]. Hasani et al. (2018) conducted a study to identify the risk of age, gender, time, pedestrian position, accident location, and vehicle type for pedestrian fatality in urban and suburban traffic collisions in Tehran and Alborz Provinces. They found that in urban roads older than 35 years; males; day time, two-way not divided roads, holidays, 4-wheeol vehicles, crossing the road from an unauthorized route were significantly associated with pedestrian fatality. However, only road design (two-way divided roads) was identified in suburban crashes to correlate with pedestrian fatalities [7]. In an earlier study, Bakhtiyari et al. (2019) evaluated human risk factors of RTIs using data from a cross-sectional study in Iran. They included all road crash data of five main suburban roads from August to February 2015. Over speeding, not warning a seat belt, reckless overtaking, fatigue and drowsiness, and exceeding the speed limit were determined to be the most important human factors affecting traffic-related deaths [8].

All the studies mentioned above indicate sparse information about risk factors related to crash severity in Iran. Furthermore, it should be considered that we are at the beginning of the United Nations Decade of Action for Road Safety 2021–2030, which emphasizes the importance of taking a holistic approach to road safety [9]. Therefore, it is crucial to know where we are, the situation where the field is, and identify what research will be essential for further progress in the future. Therefore, a comprehensive investigation of the epidemiological features of RTIs in all categories of possible risk factors, namely crash scene, vehicle, driver, passenger, and pedestrian characteristics, seems to be a vital concern. In this regard, the primary objective of the present study is to make integrated analyses to identify the main factors that affect road crash severity. To accomplish this goal and address questions on the effects of crash scene, vehicle, driver, passenger, and pedestrian characteristics, the data of a comprehensive study at the national level was used. The findings gained by this study will be helpful information that stakeholders in road safety can use to create effective countermeasures against severe and fatal crashes.

In this study, logistic regression was used to classify the statistically significant risk factors for fatal traffic accidents. The use of logistic regression has been shown to be an effective and trustworthy way to identify the relationship between the dependent and independent variables in traffic accidents. Fiorentini et al. (2020) used the random under-sampling of the majority class (RUMC) resampling technique to deal with imbalanced crash databases. The authors claimed that because classification issues are usually unbalanced, a useful prediction for the minority class may be made. To create crash severity models, four different techniques including Logistic Regression, random Tree, Random Forest, and K-Nearest Neighbor were used. Eight separate models were developed both utilizing or not utilizing RUMC and one of the four machine learning techniques. F1-score, True positive rate (recall), true negative rate, false positive rate, accuracy, precision, and the confusion matrix were calculated to evaluate the efficacy of the various models. This study looked at a dataset of 6,515 crashes that occurred on roads and at crossings in Great Britain from 2005 to 2018. In terms of predictive power, the RUMC-based methods outperformed the algorithms created utilizing the unbalanced dataset. Concerning overall accuracy, the RUMC-Logistic Regression (62.53%) outperformed the RUMC-Random Forest (56.14%), Random Tree (50.97%), and RUMC-K-Nearest Neighbor (48.47%) [10]. In a case study, Olayode et al. (2021) found that in predicting the traffic flow at a four-way road intersection, an artificial neural network trained by a particle swarm optimization model performed better than a heuristic Artificial Neural Network model. Moreover, due to their superior testing results, both models were sufficiently reliable in predicting traffic flow [11].

In one of the recent studies, Mohanty et al. (2022) examined the use of artificial neural network and binary logistic regression for modeling crash severity by looking at the role of cars (both as perpetrator & victim). When using the cut-off value equal to 0.5, the binary logistic regression effectively predicted about 75% of outcomes. The number of crashes involved in a particular offender and victim pair crash, the type of validation method used, and the hidden layer used for the study considering different sigmoid activation functions all had a substantial impact on the artificial neural network method’s accuracy. ROC curves showed that artificial neural network could correctly forecast 75% of the outcomes. By removing any pairs of vehicles that are present or that have appeared infrequently, this percentage could be increased. Based on a comparison of the two approaches’ advantages and disadvantages, binary logistic regression was found to be superior overall. Its only drawback was a lack of applicability when there was a weak correlation between the dependent variable and its predictors. However, the artificial neural network approach is unconstrained by these restrictions due to its machine-learning nature. Using more input data, it delivers predictions with more precision [12].

The following is how the paper is organized: A description of the data and research variables is offered after an overview of the relevant investigations. The findings of both the basic and multiple logistic models are then analyzed and explained, along with the management of missing data and descriptive statistics for these variables. A few closing remarks are then offered.

## Materials and methods

### Data collection and description of variables

**Fig. 1:**
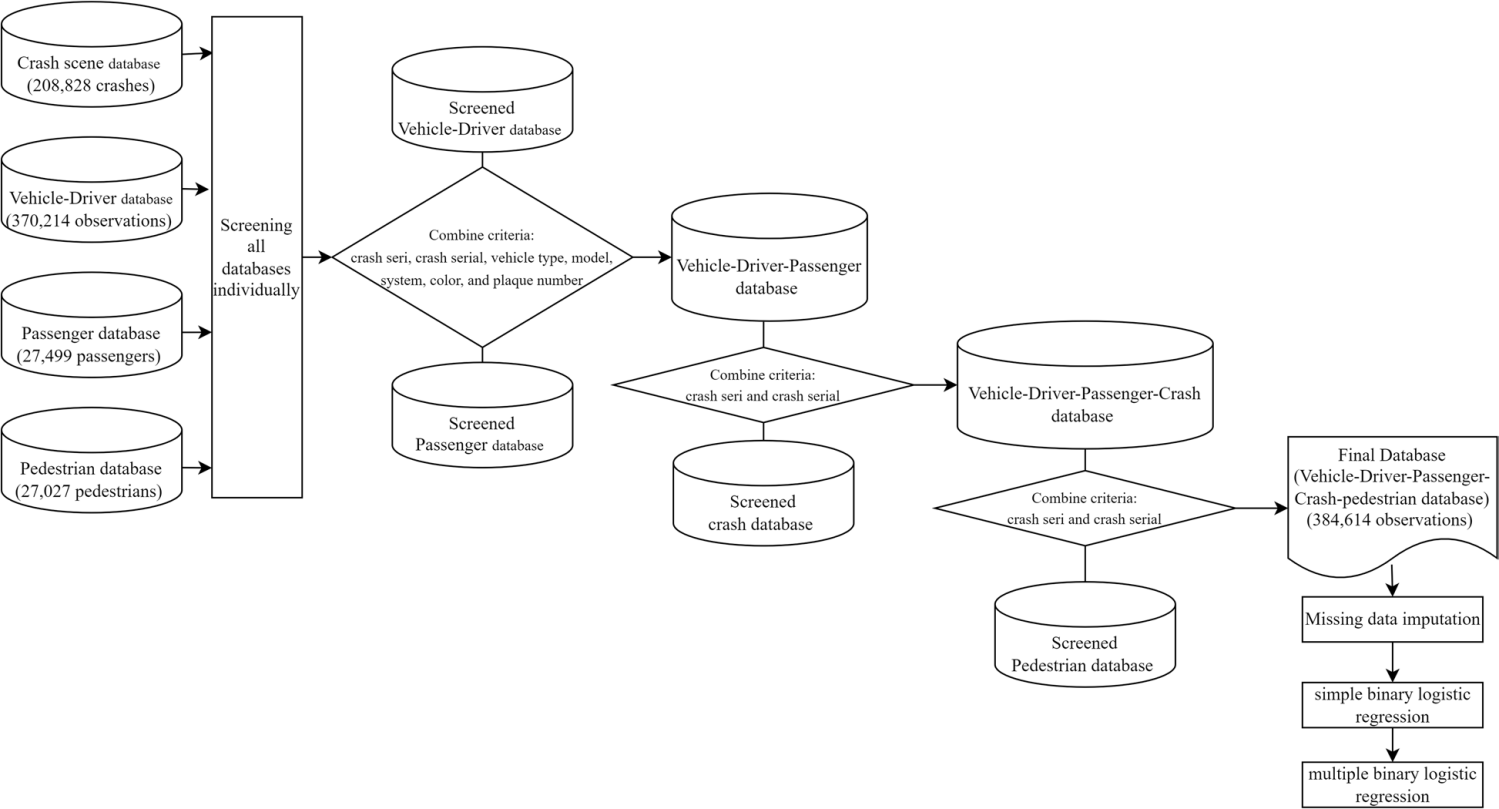
Flowchart of dataset preparation for modeling the contributing factors of fatal crashes

Reliable and expanded data collection is crucial to derive sound conclusions. In Iran, Integrated Road Traffic Injury Registry System (IRTIRS) [13] is a comprehensive reference for a crash database. This multi-method study is supported by the World Health Organization, the Iranian Ministry of Health, the Iranian Traffic Police, and the Iranian Forensic Medicine Organization. The development of IRTIR is a national research project started with 2017with the aim of developing an integrated registration of traffic accidents in Iran. In cooperation with other interested organizations, the Ministry of Health and Medical Education (MOHME) and the Road Traffic Injury Research Center of Tabriz University of Medical Sciences decided to develop IRTIR to create an integrated data recording system. Experts fill in reports in five main sections: crash scene (crash type, time, lighting status, weather, etc.), vehicle (vehicle type, color, maneuver, etc.), driver (age, gender, license, etc.), passenger (age, gender, injured organ, etc.) and pedestrian (age, gender, injured organ, etc.). This study covers all accidents in one year (March 2015—March 2016), in which 384,614 road traffic crashes were recorded on all roads in Tehran, Isfan, Fras, Razavi Khorasan, Khuzestan, and East Azerbaijan provinces, the six most populated provinces in Iran. Figure 1 presents a flowchart of dataset preparation for modeling the contributing factors of fatal crashes.

The IRTIRS provides information in four different categories in separated files. The crash database contained details of 208,828 crashes. The crash severity in this database considers three categories: property damage, injury, and fatality. Based on the study purpose, severity data are classified into two categories: (1) damage or injury and (2) fatality. The variable of severity, hence, in this study, is binary.

Additionally, the IRTIRS crash database contains the road name where the crash occurred and its type (alley, main street, side street, main road, side road, rural road, freeway, and expressway). Road names were searched manually in Google Maps to ensure correct recorded road types. In case of any doubt, the information about that particular road was asked traffic police officers. The vehicle-driver database had 370,214 observations, dropping six repeated cases led to the final target database with 370,208 observations. Overall, 27,499 recorded passengers and 27,027 pedestrians were in the following two databases.

After screening all four databases individually, they were combined to make a final master database. Initially, vehicle-driver and passenger databases were combined using crash seri, serial, vehicle type, model, system, color, and plaque number to make a vehicle-driver-passenger database. Subsequently, crash and pedestrian information was added to vehicle-driver-passenger using crash seri and serial number. In the pedestrian combing phase, the data of 94 pedestrians were removed since these pedestrians could not be matched with the diver who struck them. The final database entailed 384,614 observations: 323,884 of which contained crash-vehicle information, 26,337 with crash-vehicle- passenger, 26,264 with crash-vehicle–pedestrian, 153 with crash-vehicle-passenger-pedestrian, 10 with crash-passenger-pedestrian, 531with crash-passenger, 458 with crash-pedestrian, one with pedestrian-passenger, 6,427 with crash, 25 with vehicle, 477 with passenger, and 47 with pedestrian information. All variable descriptions and categories are detailed in Table 1. In this table, the original categories of each variable, along with the modified ones, are also presented.

**Table 1:** Variables description in Iranian Integrated Road Traffic Injury Registry System (2015–2016) – Crash database

Original variable name	Original variable levels	Modified variable name	Modified variable levels
Accident date, date format	NA	Crash season	spring, summer, fall, winter
		Crash day	weekend, weekday
Crash seri, cont	NA	NA	NA
Crash serial, cont	NA	NA	NA
Crash type	property damage, injury, death	Crash severity	non-fatal, fatal
Light status	day, night, twilight, dawn	Lightning status	Day, night, twilight/dawn
Air status	clear, rainy, cloudy, snowy, foggy, stormy, dusty	Weather	clear/cloudy, foggy/stormy/dusty, rainy, snowy
Scene status	scene without exchanged details, scene with exchanged details	NA	NA
Distance from nearest police station (km), cont	NA	NA	NA
Agent status	stayed at the scene, left the scene, had been transformed to a hospital, had died at the scene	NA	NA
Zone type	smooth, rough, mountainous	NA	NA
Intersection control	no control, traffic Police, traffic light, yield sign, stop sign, barbed gate, other	NA	NA
Road lane line marking	broken line, no line, single solid line, double solid line	NA	NA
Road material	sand-soil, asphalt	NA	NA
Land use	residential, commercial, nonresidential, agricultural, industrial, recreational, pedagogical, other	NA	residential, nonresidential, multipurpose area
Officer code, cont	NA	NA	NA
Crash mechanism	vehicle to vehicle, vehicle to multiple vehicle, vehicle to pedestrian, vehicle to livestock, vehicle to motorcycle, vehicle to fixed object, rollover, fire, vehicle to rider, pedestrian fall, bicycle to pedestrian, motorcycle to motorcycle, motorcycle to bicycle, motorcycle to pedestrian, off-road, vehicle to bicycle, vehicle to stopped vehicle, passenger thrown, multiple crash	NA	**single-vehicle crash:** rollover/ fire/ vehicle to fixed object/ vehicle to livestock/ off-road/ vehicle to stopped vehicle/ passenger thrown/ **multiple-vehicle crash:** vehicle to vehicle**/** vehicle to multiple vehicle/ multiple crash **involving vulnerable road user crash**: vehicle to pedestrian/ vehicle to motorcycle/ vehicle to rider/ pedestrian fall/ bicycle to pedestrian/ motorcycle to motorcycle/ motorcycle to bicycle/ motorcycle to pedestrian/ vehicle to bicycle
Road view obstruction	no, moving vehicle, tree/ shrub, building/ Kiosk, stopped vehicle, vertical curve, slop, hill, sunlight, fog/ smoke, front vehicle beam, sandstorm, sign, blizzard, frozen glass	NA	no, yes
Crash position	riding lane, refuge, roadside, off-road, road shoulder	Crash position in riding lane	no, yes
Road surface	dry, wet, frosty, sand-oil, muddy, greasy & oily	Dry road surface	no, yes
Road geometric design	straight, squad curve, horizontal curve, vertical curve	Curved geometric design	no, yes
Vehicle factor	no, lightning system, brake system, lack of wiper, smooth tire, steering system, lack of tire chains at emergent, suspension system	NA	no, yes
Human factor	no, hasty driving, ignoring the traffic regulations, fatigue and drowsiness, failure to detect yield, unfamiliar route, drunken driving, unstable load restraint, old age, willful misconduct, drug abuse, defected organ	NA	no, yes
First cause	*need for more driver training, lack of personal and social responsibility*, failure of road officials in repairing road defects, failure of road construction companies to install signs, lack of timely snow removal by the Ministry of Roads	NA	more training, irresponsibility, *more training & irresponsibility*, failure of organs, multiple factors
Prior cause	*hasty driving, lack of attention to driving*, lack of sufficient skills in driving, fatigue and drowsiness, lack of skill in diagnosing traffic situation, slippery or tarred road surface, technical defects of the vehicle, obstacles and bumps, drunken driving, lack of skills in diagnosing road conditions, lack of road position detection, catching the opportunity, sharp horizontal curve, heavy snowfall or rain, physical weakness, storms and dust, sharp slope, road position and taken speed mismatch, mental and nervous system defect, visual and auditory senses defect, skeleton defect	NA	hasty driving, lack of attention to driving, *hasty driving & lack of attention to driving*, lacked skill, other
Direct cause	ignoring the traffic regulations, delay in sighting, over speeding, escaping the crash in a wrong way	NA	ignoring the traffic regulations, delay in sighting, over speeding, escaping the crash in a wrong way or multiple factor
Collision type	front bumper to front bumper, front bumper to back bumper, front bumper to right side, front bumper to left side, left side to right side, front bumper to fixed object, back bumper to right side, back bumper to left side, left side to left side, right side to right side, right side to fixed object, left side to fixed object, back bumper to fixed object, other	NA	head-on collision, rear-end collisions, T-bone collision, side-swipe collision, fixed-object collision, other
Province	Tehran-bozorg, Tehran East, Tehran West, Isfahan, Khorasan Razavi, Khouzestan, Fars, East Azerbaijan	NA	Tehran, Isfahan, Khorasan Razavi, Khouzestan, Fars, East Azerbaijan
Crash longitude coordinate (degree), cont	NA	NA	NA
Crash latitude coordinate (degree), cont	NA	NA	NA
Crash commuting area	urban, suburban, rural road, exclusive urban area, exclusive suburban area	NA	NA
Road type^a^	freeway, expressway, main road, main street, side street, side road, rural road, straight road, alley, square, intersection, main arterial street, boulevard, entrance ramp, side arterial street, bridge, one-sided, horizontal curve, other	NA	freeway, expressway, main road, side road, rural road, main street, side street, alley
Road shoulder	unpaved, soil, asphalt	NA	NA
Road shoulder width (m), cont	NA	NA	NA
Road beginning, str	NA	NA	NA
Road end, str	NA	NA	NA
Road length(km), cont	NA	NA	NA
Road width (m), cont	NA	NA	NA
Road design	separated two-way road, unseparated two-way road, one-way road	NA	NA
Road defect	no, defect of vertical traffic signs, narrow passage, defect of horizontal traffic signs, passage marking defect, passage lightning defect, lack of soil shoulder and parking, lack of safety guard next to the passage, bump obstacle, sharp arc, level difference between asphalt and shoulder, asphalt surface defects, road collapse, slippery road surface, non-standard latitudinal and longitudinal slopes, non-standard guard next to the passage	NA	no, pavement defects, signs defects, geometric defects, lightning defects
Road permitted speed (Km/h), cont	NA	NA	< = 30, 30–50, 50–60, 60–80, 80–95, 95–110, 110–120
Road repairing status	no repairs, under repairing without adequate signs, under repairing with adequate signs	NA	no, yes
Vehicle type	passenger car, motorcycle, pickup truck, lorry, taxi,, two trunk, mini-trunk, bus, minibus, bicycle, ambulance, tractor, constructional tools, agricultural tools, police car, semi-trailer, trailer, trail, lpg (Liquefied Petroleum Gas) tanker trailer, auto camping, fire truck	Vehicle type based on European Commission	L: tricycle/ bicycle/motorcycle, M_L: passenger < 9 P, M_H: passenger > 9 P, N_L: goods < = 6 T, N_H: goods > = 6 T, T: agricultural/ constructional tools
		Vehicle type based on previous studies	light, heavy, tricycle/ bicycle/motorcycle
Vehicle system, cont	1007 values indicating vehicle’s brand such as: BMW, Benz, Toyota, Hyundai, pride, peugeot, etc	NA	NA
Vehicle system ID, cont	2394 values indicating vehicle’s model such as: X4, C200, Corolla, Sonata, 141, 2008, etc	NA	NA
Vehicle company, cont	131 values indicating manufacturer company such as: BMW, Benz, Toyota, Hyundai, Saipa, Iran Khodro, etc	NA	NA
Vehicle parent company, cont	94 values indicating owner organization such as: Post office, Ministry of foreign affairs, bus service, etc	NA	NA
Vehicle safety equipment	no, abs, airbag, abs & airbag	NA	NA
Vehicle color	white, silver, graphite gray, black, blue, green, yellow, cream, red, orange, brown, dark blue, gray, purple, pink	NA	low risk, high risk
Vehicle plaque number, str	NA	NA	NA
Vehicle plaque serial, str	NA	NA	NA
Vehicle year produced, cont	NA	Vehicle life	less than 5yrs, 5 to 9 yrs, 10 to 14 yrs, 15 and more than 15yrs
Vehicle plaque description	38 values indicating plaque type such as: personal regional, free zones, governmenta, etc		personal regional, other
Vehicle moving direction	N-S, E-W, W-E, S–N, S-W, N-E, W–N, E-S, N-W, S-E, E-N, W-S	NA	cardinal direction, ordinal direction
Vehicle maneuver	moving ahead, turning left, stopping on the road, turning, turning right, moving backward, stopping outside of the road, sudden starting, sudden stopping, overtaking, spiral movement	NA	moving forward, turning, stopping on the road, moving backward, overtaking, other
Remained effect	no, detached vehicle parts, asphalt damage, poured oil, other	NA	NA
Driver first name, str	NA	NA	NA
Driver last name, str	NA	NA	NA
Driver national ID. cont	NA	NA	NA
Driver fault status	at fault, not at fault	NA	NA
Driver gender	male, female	NA	NA
Driver education	illiterate, literacy, elementary, cycle, middle school, diploma, B.Sc., A.Sc., M.Sc., PhD, hozavi	Driver education based on ISCED	isced0, isced1, isced2, isced3, isced5, isced6
		Driver education based on previous studies	illiterate, primary, nonacademic, academic
Driver job	self-employed, employee, jobless, housewife, military man, laborer, student, university student, driver, soldier, other	NA	jobs with high economic status, jobs with middle economic status, jobs with low economic status
Driver age (yrs), cont	NA	NA	child, adult, old
Type of driving license	class A, class B, class C, B1, B1-new, B2, B1-Temporary, B2-temporary, military, special, foreign, international, motorcycle, tractor, not seen, no license	NA	class A, class B, class C, motorcycle, no license
Driver license ID, cont		NA	NA
Driver injury type	not-injured, injured, dead	NA	NA
Driver total reason	lack of attention to the front, failure to yield right-of-way, failure to maintain vehicle control, changing direction abruptly, moving backward in reverse gear, failure to longitudinal distance control, deviation to the left, driving in the wrong direction, making incorrect left turn, violation of safe speed, failure to latitudinal distance control, Sudden opening of the vehicle door, deviation to the left due to overtaking, running red light, passing the prohibited place, turning in the prohibited place, vehicle technical defect, over speeding, lack of skills in driving, persistent technical defects of the vehicle, deviation to the right, violation of load regulations, violation of item 4 of the road safety, towing incorrectly, pedestrian fault, other	NA	lack of attention to the front, failure to yield right-of-way, failure to maintain vehicle control, changing direction abruptly, moving backward in reverse gear, failure to longitudinal distance control, other
Driver seat belt usage status	used, not used	NA	NA
Driver reaction	no reaction, brake, deviation to the right, get out of the car	NA	NA
Driver Judiciary cause	carelessness, unconscious, lack of driving skills, violation of the law	NA	carelessness, other
Driver misconduct	failure to yield right-of-way, failure to yield right-of-way, over speeding, failure to distance control while overtaking, running red light, passing the prohibited place, illegal overtaking, turning left or right in the prohibited place, turning in the prohibited place, drunken driving, lack of safety equipment for the season, not turning on the lights from sunset to sunrise, not using glasses while driving, defective vehicle lighting system at night, demonstrative movement, spiral movement, crossing the sidewalk	NA	spiral movement, over speeding, other
Passenger first name, str	NA	NA	NA
Passenger last name, str	NA	NA	NA
Passenger gender	male, female	NA	NA
Passenger education	literate, literacy, elementary, cycle, middle school, diploma, B.Sc., A.Sc., M.Sc	Passenger education based on ISCED	isced0, isced1, isced2, isced3, isced5
		Passenger education based on previous studies	illiterate, primary, non-academic, academic
Passenger job	self-employed, employee, jobless, housewife, military man, laborer, student, university student, driver, soldier	NA	jobs with high economic status, jobs with middle economic status, jobs with low economic status
Passenger age (yrs), cont	NA	NA	child, adult, old
Passenger injury type	injured, dead	NA	NA
Passenger seat belt usage status	used, not used	NA	NA
Passenger injured organ	head and face, neck, hand and arm, chest and abdomen, legs, back, right leg, left leg, pelvis, right hand, left hand, shoulder, skull, forehead, right eye, eyes, left eye, other	Passenger injured organ based on ICD10 codes	S0-S1 (head), S4-S6 (upper limb), S2-S3 (trunk), S7-S9 (lower limb), other
Passenger fault status	at fault, not at fault	NA	NA
Passenger total reason	passenger fault, passenger total reason1, passenger total reason2	NA	NA
Pedestrian first name, str	NA	NA	NA
Pedestrian last name, str	NA	NA	NA
Pedestrian injury type	injured, dead	NA	NA
Pedestrian clothes color	light, dark	NA	NA
Pedestrian status	NA	NA	low-risk, moderate-risk, high-risk
Pedestrian injured organ	head and face, neck, hand and arm, chest and abdomen, legs, back, right leg, left leg, pelvis, right hand, left hand, shoulder, skull, forehead, right eye, eyes, left eye, other	Pedestrian injured organ based on ICD10 codes	S0-S1 (head), S4-S6 (upper limb), S2-S3 (trunk), S7-S9 (lower limb), other
Passage utilities	no, no need, zebra crossing, footbridge, underpass	NA	no, yes
Passage place status	allowed, not allowed	NA	NA
pedestrian gender	male, female	NA	NA
Pedestrian national ID, cont	NA	NA	NA
Pedestrian education	illiterate, literacy, elementary, cycle, middle school, diploma, A.Sc., hozavi, B.Sc., M.Sc., doctoral degree, PhD	Pedestrian education based on ISCED	isced0, isced1, isced2, isced3, isced5, isced6
		Pedestrian education based on previous studies	illiterate, primary, non-academic, academic
Pedestrian job	self-employed, employee, jobless, housewife, military man, laborer, student, university student, driver, soldier	NA	jobs with high economic status, jobs with middle economic status, jobs with low economic status
Pedestrian age (yrs), cont	NA	NA	child, adult, old
Pedestrian fault status	at fault, not at fault	NA	
Pedestrian total reason	not using the designated crossings, sudden entrance to the road, not using the designated crossings on highways and main streets, crossing the freeways’ fence and trees and shrubs, running the red light	NA	unsafe crossings in urban areas, unsafe crossings in sub-urban area
Pedestrian transfer type	ambulance, crossing vehicle	NA	NA
Pedestrian judiciary cause	carelessness, negligence, non-compliance with government systems, lack of driving skills	NA	carelessness, other

Dotted lines indicate there are several types of classifications. The underlined phrases with equal styles or fonts represent the same classification of original variables and the modified ones

*str.* String, *cont.* Continuous, *NA* Not applicable, *yrs* Years, *P* Person, *T* Tone

^a^Phrases without underline considered as missing

### Missing data management

Numerous statistical methods have been proposed to manage missing data [14]. In epidemiological studies, complete case analyses (CCA) and multiple imputations are standard approaches. The use of comprehensive case analyses, which only take in respondents to all variables for the intended analysis, is more common because of its simplicity and being the default of most statistical soft wares. Despite the advantages mentioned, taking a reduced and unrepresentative sample, leading to lower power and possibly biased results, can be considered this method’s major pitfalls [15]. Besides, the accuracy of this method strongly relies on assumptions concerning missing-data mechanisms, frequently needing strict missing completely at random (MCAR) assumptions. Based on this assumption, there is no relationship between either observed and unobserved variables for a given subject and the probability of a variable being missing for that subject [16].

Another alternative approach to managing missing data is the imputation method [17]. Generally, there are two imputation methods: single (SI) and multiple imputations (MI). In a SI, the imputed value is determined using a specific rule. There are several forms for the SI, including using the last observed value, using the mean, and using the data with the highest frequency. In general, the SI method is not recommended due to the need for assumptions that are often unrealistic and lead to underestimation or overestimation of the *P* value [15]. The other imputation method, MI which has gained popularity in the past few years, was developed to address the CCA drawbacks and SI. MI is a three-stage statistical process limiting uncertainty about missing values by calculating various possibilities or imputations.

- Stage 1. Multiple copies of the database are created in which the missing values are replaced. The imputed values are drawn based on the observed values and from the appropriate statistical models and the previous distribution. Each entirely imputed database is different from the other one.
- Stage 2. The analysis is performed on each complete database, which leads to an estimate of the parameter and the corresponding standard errors for each dataset.
- Stage 3. At this stage, the results obtained from the second stage are combined into a final result [15].

In the multiple imputation method, all participants can present in the analysis and may increase parameter estimation accuracy while reducing bias [18, 19]. MI is used in this paper in which each variable with missing values is imputed ten times.

### Statistical analysis

In a primary descriptive analysis, the data were described as frequencies (percentages) for categorical variables and mean ± SD (standard deviation) for continuous ones. Simple logistic regression models were performed to identify the potential explanatory variables affecting fatal crash likelihood, considering the dependent variable (crash severity: injury or property damage only crashes (Y = 0) and fatal crashes (Y = 1)) in the individual databases. Both CCA and MI in separate and combined databases were considered in simple logistic regression analysis. Since there was no significant difference in the intensity and direction of estimated odd ratios, the multiply imputed and combined database was considered the final database. Given the relatively low number of passenger and pedestrian crashes, the present study only focuses on the explanatory variables of crash and vehicle level. However, descriptive statistics have been generated for these databases; only two binary variables have been considered in the multiple analysis to indicate the presence or absence of the passenger or pedestrian in the desired crash. The effect size was identified with 95% confidence intervals for all variations. All analyses used Stata software (version 14.0; StataCorp, College station, Texas, USA).

## Results and discussion

From all variables in Table 1, those affecting the incidence of fatal traffic accidents were considered explanatory variables. Given the highly invalid data concerning distance from the nearest police station, crash longitude coordinate, crash latitude coordinate, road shoulder width, road length, and road width, this study has not considered these variables in further analyses. There were also some identifying variables in Table 1, namely: crash seri, crash serial, officer code, road name, police station, road beginning, road end, vehicle system, vehicle system ID, vehicle company, vehicle parent company, vehicle plaque number, vehicle plaque serial, driver first name, driver last name, driver national ID, driving license ID, passenger first name, passenger last name, passenger national ID, pedestrian first name, pedestrian last name, pedestrian national ID, which were just used either for producing unique linkage ID to combine different databases or finding out whether the databases were correctly combined. In addition, scene status, driver reaction, and driver injury type were removed from simple and multiple analyses because these variables are outcomes. Table 2 offers an explanatory variables summary. From 208,828 crashes recorded in the crash database, 2,237 (1.07%) were fatal. Details about all other explanatory variables have been presented in Table 2. In the case of defining modified levels for a variable, the statistics have been described based on the modified levels.

**Table 2:** Explanatory variables summary in Iranian Integrated Road Traffic Injury Registry System (2015–2016) – crash database

Variable	Viable level	Total crashes n (%)	Fatal crashes n (%)
Crash day	weekday	150,730 (98.97)	1,561 (1.03)
	weekend	55,861 (98.80)	676 (1.20)
Lightning	missing	7,076 (99.34)	47 (0.66)
	Day	141,247 (99.15)	1,206 (0.85)
	night	52,094 (25.35)	850 (1.61)
	twilight/dawn	6,174 (3.02)	134 (2.12)
Weather	missing	7,204 (3.45)	44 (0.61)
	clear/cloudy	197,603 (94.62)	2,130 (1.08)
	foggy/stormy/dusty	362 (0.17)	6 (1.66)
	rainy	3,165 (1.52)	51 (1.61)
	snowy	494 (0.24)	6 (1.21)
Scene status	scene without exchanged details	11,329 (5.43)	113 (1.00)
	scene with exchanged details	197,499 (94.57)	2,124 (1.08)
Agent status	missing	489 (0.23)	4 (0.82)
	stayed at the scene	200,887 (96.20)	1,866 (0.93)
	had left the scene	2,261 (1.08)	64 (2.83)
	had been transformed to a hospital	5,005 (2.4)	123 (2.46)
	had died at the scene	186 (0.09)	180 (96.77)
Zone type	missing	2,088 (1.00)	20 (0.96)
	smooth	203,999 (97.69)	2,104 (1.03)
	rough	745 (0.36)	36 (4.83)
	mountainous	1,996 (0.96)	77 (3.86)
Intersection control	missing	64,571 (30.92)	676 (1.05)
	No	50,807 (24.33)	677 (1.33)
	Yes	93,450 (44.75)	884 (0.95)
Road lane line marking	missing	70,701 (33.86)	955 (1.35)
	broken line	28,902 (13.84)	219 (0.76)
	no line	100,575 (48.16)	861 (0.86)
	single solid line	7,893 (3.78)	192 (2.43)
	double solid line	757 (0.36)	10 (1.32)
Road material	missing	1,764 (0.84)	20 (1.13)
	sand-soil	206,548 (98.91)	2,175 (1.05)
	asphalt	516 (0.25)	42 (8.14)
Land use	missing	3,131 (1.5)	28 (0.89)
	residential	126,501 (60.58)	661 (0.52)
	nonresidential	34,464 (16.50)	1,144 (3.32)
	other uni-purpose areas	18,818 (9.01)	292 (1.55)
	multipurpose area	25,914 (12.41)	112 (0.43)
Crash mechanism	missing	2,474 (1.19)	10 (0.40)
	single-vehicle crash	25,431 (12.18)	741 (2.91)
	multiple-vehicle crash	109,265 (52.32)	581 (0.53)
	involving vulnerable road users crash	71,658 (34.31)	905 (1.26)
View obstruction	missing	8,401 (4.02)	67 (0.80)
	no	197,135 (94.40)	2,107 (1.07)
	yes	3,292 (1.58)	63 (1.91)
Crash position in riding lane	missing	8,405 (4.02)	70 (0.83)
	no	5,920 (2.83)	332 (5.61)
	yes	194,503 (93.14)	1,835 (0.94)
Dry road surface	missing	8,124 (3.89)	61 (0.75)
	no	4,635 (2.22)	79 (1.70)
	yes	196,069 (93.89)	2,097 (1.07)
Curved geometric design	missing	8,496 (4.07)	67 (0.79)
	no	188,854 (90.44)	1,852 (0.98)
	yes	11,478 (5.50)	318 (2.77)
Vehicle factor	missing	6,956 (3.33)	42 (0.60)
	no	200,611 (96.07)	2,179 (1.09)
	yes	1,261 (0.60)	16 (1.27)
Human factor	missing	5,974 (2.86)	36 (0.60)
	no	46,344 (22.19)	606 (1.31)
	yes	156,510 (74.95)	1,595 (1.02)
First cause	missing	97,652 (46.76)	58 (0.06)
	more training	61,455 (29.43)	1,396 (2.27)
	irresponsibility	29,597 (14.17)	346 (1.17)
	more training & irresponsibility	19,347 (9.26)	403 (2.08)
	failure of state organs	190 (0.09)	7 (3.68)
	multiple factors	587 (0.28)	27 (4.60)
Prior cause	missing	97,677 (46.77)	59 (0.06)
	hasty driving	51,299 (24.57)	893 (1.74)
	lack of attention to driving	37,173 (17.80)	738 (1.99)
	hasty driving & lack of attention to driving	15,558 (7.45)	225 (1.45)
	lacked skill	3,763 (1.80)	123 (3.27)
	other	3,358 (1.61)	199 (5.93)
Direct cause	missing	97,699 (46.78)	58 (0.06)
	regulation	88,782 (42.51)	1,603 (1.81)
	delay in sighting	14,915 (7.14)	335 (2.25)
	overspending	5,960 (2.85)	209 (3.51)
	Escaping crash in a wrong way or multiple factor	1,472 (0.70)	32 (2.17)
Collision type	missing	50,439 (24.15)	954 (1.89)
	rear-end collisions	24,142 (11.56)	490 (2.03)
	T-bone collision	59,238 (28.37)	262 (0.44)
	head-on collision	48,528 (23.24)	341 (0.70)
	side-swipe collision	20,846 (9.98)	84 (0.40)
	fixed-object collision	5,635 (2.70)	106 (1.88)
Crash province	Isfahan	44,981 (21.54)	573 (1.27)
	Fras	19,111 (9.15)	365 (1.91)
	Khorasan Razavi	23,895 (11.44)	380 (1.59)
	Khouzestan	20,607 (9.87)	420 (2.04)
	East Azerbaijan	11,607 (5.56)	94 (0.81)
	Tehran	88,627 (42.44)	405 (0.46)
Commuting area	missing	1,472 (0.70)	9 (0.61)
	urban	170,090 (81.45)	720 (0.42)
	suburban	32,824 (15.72)	1,309 (3.99)
	rural road	3,467 (1.66)	167 (4.82)
	exclusive urban area	511 (0.24)	7 (1.37)
	exclusive suburban area	464 (0.22)	25 (5.39)
Road type	missing	2,013 (0.96)	7 (0.35)
	freeway	4,792 (2.29)	130 (2.71)
	expressway	32,124 (15.38)	357 (1.11)
	main street	117,309 (56.17)	437 (0.37)
	side street	13,199 (6.32)	69 (0.52)
	main road	27,672 (13.25)	763 (2.76)
	side road	7,339 (3.51)	302 (4.12)
	rural road	3,237 (1.55)	155 (4.79)
	alley	1,143 (0.55)	17 (1.49)
Road shoulder	missing	8,361 (4.00)	68 (0.81)
	asphalt	175,670 (84.12)	1,094 (0.62)
	soil	14,477 (6.93)	610 (4.21)
	unpaved	10,320 (4.94)	465 (4.51)
Road design	missing	7,230 (3.46)	48 (0.66)
	separated two-way road	44,316 (21.22)	593 (1.34)
	unseparated two-way road	104,386 (49.99)	1791 (1.71)
	one-way road	52,896 (25.33)	877 (1.66)
Road defect	missing	7,292 (3.49)	49 (0.67)
	no	192,609 (92.23)	1,859 (0.97)
	pavement/ lightning defects	4,118 (1.97)	100 (2.43)
	signs defects	2,383 (1.14)	84 (3.52)
	geometric defects	800 (0.38)	44 (5.50)
	multiple defects	1,626 (0.78)	101 (6.21)
Permitted speed (Km/h), cont	missing	97,298 (46.59)	0 (0.00)
	mean ± SD	51.59 ± 25.15	NA
Permitted speed	missing	97,335 (46.61)	52 (0.05)
	< = 30	32,835 (15.72)	250 (0.76)
	30–50	38,281 (18.33)	417 (1.09)
	50–60	18,055 (8.65)	348 (1.93)
	60–80	9,832 (4.71)	277 (2.82)
	80–95	5,556 (2.66)	382 (6.88)
	95–110	5,582 (2.67)	409 (7.33)
	110–120	1,352 (0.65)	102 (7.54)
Road repairing status	missing	98,216 (47.03)	69 (0.07)
	no	109,784 (52.57)	2,132 (1.94)
	yes	828 (0.40)	36 (4.35)
Vehicle type	missing	1,843 (0.50)	21 (1.14)
	light	255,980 (69.14)	1,496 (0.58)
	heavy	58,185 (15.72)	1,028 (1.77)
	tricycle/ bicycle/motorcycle	54,200 (14.64)	633 (1.17)
Vehicle safety equipment	missing	6,174 (1.67)	42 (0.68)
	no	302,909 (81.82)	2,776 (0.92)
	yes	61,125 (16.51)	360 (0.59)
Vehicle color	missing	86,038 (23.24)	964 (1.12)
	low risk	159,258 (43.02)	1,347 (0.85)
	high risk	124,912 (33.74)	867 (0.69)
Vehicle year produced, cont	missing	247,867 (66.95)	0 (0.00)
	mean ± SD	7.61 ± 4.41	NA
Vehicle life (yrs), cont	less than 5yrs	40,748 (11.01)	666 (1.63)
	5 to 9 yrs	47,552 (12.84)	862 (1.81)
	10 to 14 yrs	24,011 (6.49)	384 (1.60)
	15 and more than 15yrs	10,030 (2.71)	352 (3.51)
Vehicle plaque description	missing	4,196 (1.13)	98 (2.34)
	personal regional	269,819 (72.88)	1,737 (0.64)
	other	96,193 (25.98)	1,343 (1.40)
Vehicle moving direction	missing	187,745 (50.71)	75 (0.04)
	cardinal direction	177,321 (47.9)	3,066 (1.73)
	ordinal direction	5,142 (1.39)	37 (0.72)
Vehicle maneuver	missing	190,031 (51.33)	76 (0.04)
	moving forward	159,030 (42.96)	2,871 (1.81)
	turning	12,486 (3.37)	103 (0.82)
	overtaking	275 (0.07)	19 (6.91)
	moving backward	2,345 (0.63)	35 (1.49)
	stopping on the road	4,283 (1.16)	46 (1.07)
	other	1,758 (0.47)	28 (1.59)
Vehicle remained effect	missing	11,346 (6.35)	139 (1.23)
	asphalt damage	5,098 (2.85)	159 (3.12)
	detached parts	98,032 (54.84)	1,716 (1.75)
	poured oil	1,034 (0.58)	21 (2.03)
	other	55,774 (31.20)	552 (0.99)
	multiple-effect	7,462 (4.17)	502 (6.73)
Driver fault status	missing	17 (0.00)	0 (0.00)
	at fault	203,157 (54.88)	2,176 (1.07)
	not at fault	167,034 (45.12)	1,002 (0.60)
Driver gender	missing	1,143 (0.31)	22 (1.92)
	male	334,122 (90.25)	3,044 (0.91)
	female	34,943 (9.44)	112 (0.32)
Driver education	missing	52,555 (14.2)	787 (1.5)
	illiterate	5,606 (1.51)	70 (1.25)
	primary	17,471 (4.72)	170 (0.97)
	nonacademic	264,836 (71.54)	2,027 (0.77)
	academic	29,740 (8.03)	124 (0.42)
Driver job	missing	95,748 (25.86)	1,662 (1.74)
	jobs with high economic status	225,475 (60.9)	1,343 (0.60)
	jobs with middle economic status	23,252 (6.28)	108 (4.09)
	jobs with low economic status	25,733 (6.95)	165 (6.41)
Driver age (yrs), cont	missing	24,782 (6.69)	0 (0.00)
	mean ± SD	36.47 ± 12.30	NA
	child	4,306 (1.16)	55 (1.28)
	adult	322,358 (87.07)	2,616 (0.81)
Type of driving license	missing	28,454 (7.69)	419 (1.47)
	class A	30,272 (8.18)	454 (1.50)
	class B	127,669 (34.49)	514 (0.40)
	class C	160,917 (43.47)	1,585 (0.98)
	motorcycle	10,030 (2.71)	23 (0.23)
	no license	12,866 (3.48)	183 (1.42)
Driver injury type	missing	2,955 (0.80)	33 (1.12)
	not-injured	312,673 (84.46)	1,499 (0.48)
	injured	53,444 (14.44)	511 (0.96)
	dead	1,136 (0.31)	1,135 (99.91)
Driver total reason	missing	167,553 (45.26)	1,006 (0.60)
	lack of attention to the front	64,610 (17.45)	808 (1.25)
	failure to yield right-of-way	39,651 (10.71)	152 (0.38)
	failure to maintain vehicle control	19,732 (5.33)	466 (2.36)
	changing direction abruptly	16,266 (4.39)	58 (0.36)
	moving backward in reverse gear	11,721 (3.17)	47 (0.40)
	failure to longitudinal distance control	11,070 (2.99)	21 (0.19)
	other	39,605 (10.70)	620 (1.57)
Driver seat belt usage status	missing	304,987 (82.38)	1,832 (0.60)
	used	36,400 (9.83)	667 (1.83)
	not used	28,821 (7.79)	679 (2.36)
Driver reaction	missing	346,381 (93.56)	2,788 (0.80)
	brake	3,742 (1.01)	96 (2.57)
	deviation to the right	131 (0.04)	7 (5.34)
	get out of the car	43 (0.01)	4 (9.30)
	no reaction	19,911 (5.38)	283 (1.42)
Driver Judiciary cause	missing	260,381 (70.33)	1,038 (0.40)
	carelessness	101,974 (27.55)	1,893 (1.86)
	other	7,853 (2.12)	247 (3.15)
Driver misconduct	missing	359,692 (97.16)	2,943 (0.82)
	spiral movement	5,465 (1.48)	43 (0.79)
	over speeding	2,861 (0.77)	109 (3.81)
	other	2,190 (0.59)	83 (3.79)
Passenger gender	missing	110 (0.40)	9 (8.18)
	male	15,157 (55.12)	886 (5.85)
	female	12,232 (44.48)	605 (4.95)
Passenger education	missing	15,008 (54.58)	793 (5.28)
	illiterate	840 (3.05)	77 (9.17)
	primary	678 (2.47)	42 (6.19)
	nonacademic	10,777 (39.19)	573 (5.32)
	academic	196 (0.71)	15 (7.65)
Passenger job	missing	24,951 (90.73)	1,370 (5.49)
	jobs with high economic status	675 (2.45)	17 (2.52)
	jobs with middle economic status	1,102 (4.01)	86 (0.00)
	jobs with low economic status	771 (2.86)	27 (0.00)
Passenger age (yrs), cont	missing	3,160 (11.5%)	0 (0.00)
	mean ± SD	29.42 ± 15.63	NA
	child	4,665 (16.96)	294 (6.30)
	adult	18,336 (66.68)	956 (5.21)
	elderly	1,338 (4.87)	105 (7.85)
Passenger injury type	missing	124 (0.45)	1 (0.81)
	injured	26,665 (96.97)	790 (2.96)
	dead	710 (2.58)	709 (99.86)
Passenger seat belt usage status	missing	18,930 (68.84)	977 (5.16)
	not used	7,508 (27.3)	430 (5.73)
	used	1,061 (3.86)	93 (8.77)
Passenger injured organ based on ICD10 codes	missing	15,260 (55.49)	629 (4.12)
	S0-S1 (head)	9,307 (33.84)	750 (8.06)
	S2-S3 (trunk)	2,205 (8.02)	311 (14.10)
	S4-S6 (upper limb)	2,695 (9.80)	177 (6.57)
	S7-S9 (lower limb)	2,086 (7.59)	116 (5.56)
	other	14,113 (51.32)	639 (4.53)
Passenger fault status	missing	12,732 (46.30)	948 (7.45)
	at fault	1,879 (6.83)	162 (8.62)
	not at fault	12,888 (46.87)	390 (3.03)
Passenger total reason	missing	27,319 (99.35)	1,482 (5.42)
	passenger fault	180 (0.65)	18 (10.00)
Pedestrian injury type	missing	543 (2.01)	3 (0.55)
	injured	26,038 (96.34)	46 (0.18)
	dead	446 (1.65)	445 (99.78)
Pedestrian clothes color	missing	14,807 (54.79)	245 (1.65)
	light	4,180 (15.47)	86 (2.06)
	dark	8,040 (29.75)	163 (2.03)
Pedestrian status	missing	6,425 (23.77)	132 (2.05)
	low-risk	17,330 (64.12)	209 (1.21)
	moderate-risk	3,039 (11.24)	136 (4.48)
	high-risk	233 (0.86)	17 (7.30)
Pedestrian injured organ based on ICD10 codes	missing	22,628 (83.72)	427 (1.89)
	S0-S1 (head)	2,075 (7.68)	55 (2.65)
	S2-S3 (trunk)	318 (1.18)	17 (5.35)
	S4-S6 (upper limb)	732 (2.71)	12 (1.64)
	S7-S9 (lower limb)	1,790 (6.62)	14 (0.8)
	other	5,076 (18.78)	91 (1.79)
Passage utilities	missing	24,641 (91.17)	446 (1.81)
	no	1,139 (4.21)	21 (1.84)
	yes	1,247 (4.61)	27 (2.17)
Passage place status	missing	24,528 (90.75)	462 (1.88)
	allowed	2,287 (8.46)	22 (0.96)
	not allowed	212 (0.78)	10 (4.72)
Pedestrian gender	missing	69 (0.26)	1 (1.45)
	male	17,774 (65.76)	371 (2.09)
	female	9,184 (33.98)	122 (1.33)
Pedestrian education	missing	16,778 (62.08)	267 (1.59)
	illiterate	1,124 (4.16)	29 (2.58)
	primary	858 (3.17)	19 (2.21)
	non-academic	8,087 (29.92)	177 (2.19)
	academic	180 (0.67)	2 (1.11)
Pedestrian job	missing	25,697 (95.08)	475 (1.85)
	jobs with high economic status	499 (1.85)	6 (1.20)
	jobs with middle economic status	179 (0.66)	7 (3.91)
	jobs with low economic status	652 (2.41)	6 (0.92)
Pedestrian age (yrs), cont	missing	2,923 (10.82)	NA
	mean ± SD	36.24 ± 21.75	NA
	child	5,319 (19.68)	70 (1.32)
	adult	14,454 (53.48)	230 (1.59)
	elderly	4,331 (16.02)	131 (3.02)
Pedestrian fault status	missing	620 (2.29)	6 (0.97)
	at fault	1,406 (5.20)	67 (4.77)
	not at fault	25,001 (92.50)	421 (1.68)
Pedestrian total reason	missing	26,515 (98.11)	471 (1.78)
	unsafe crossings in urban areas	377 (1.39)	13 (3.45)
	unsafe crossings in sub-urban area	135 (0.50)	10 (7.41)
Pedestrian transfer type	missing	21,618 (79.99)	429 (2.00)
	ambulance	407 (1.51)	4 (1.00)
	crossing vehicle	5,002 (18.51)	61 (1.20)
Pedestrian judiciary cause	missing	26,464 (97.92)	466 (1.76)
	carelessness	461 (1.71)	9 (1.95)
	other	102 (0.38)	3 (2.94)

*Freq.* Frequency, *Per.* Percentage, *yrs* Years, *SD* Standard deviation, *NA* Not applicable

### Analysis of the General Model

Table 3 shows each factor’s adjusted odds ratios through the final dataset based on simple and multiple logistic regression models.

**Table 3:** Simple and Multiple logistic regression models in predicting fatality based on Iranian-Integrated Road Traffic Injury Registry System (2015–2016)

Variable	Simple logistic regression		Multiple logistic regression	
	**OR (95% CI)**	***P* value**	**OR (95% CI)**	***P* value**
Passenger include				
no	reference		reference	
yes	4.94 (4.53 to 5.40)	< 0.001	4.95 (4.54 to 5.40)	< 0.001
Pedestrian include				
no	reference		reference	
yes	2.56 (1.73 to 3.78)	< 0.001	2.60 (1.75 to 3.85)	< 0.001
Crash day				
weekday	reference			
weekend	1.05 (0.97 to 1.13)	0.235		
Lightning status				
day	reference		reference	
night	1.64 (1.52 to 1.76)	< 0.001	1.64 (1.52 to 1.76)	< 0.001
twilight/dawn	1.47 (1.25 to 1.73)	< 0.001	1.48 (1.25 to 1.74)	< 0.001
Weather				
clear/cloudy	reference		reference	
foggy/stormy/dusty	0.46 (0.2 to 1.06)	0.071	0.46 (0.20 to 1.05)	0.064
rainy	1.30 (0.89 to 1.88)	0.172	1.32 (1.06 to 1.64)	0.014
snowy	0.34 (0.14 to 0.84)	0.02	0.35 (0.15 to 0.83)	0.016
Zone type				
smooth	reference			
rough	1.18 (0.88 to 1.58)	0.259		
mountainous	1.08 (0.87 to 1.34)	0.465		
Intersection control				
yes	reference		reference	
no	1.39 (1.29 to 1.51)	< 0.001	1.40 (1.29 to 1.51)	< 0.001
Line marking				
no line	reference		reference	
broken line	1.32 (1.18 to 1.49)	< 0.001	1.36 (1.21 to 1.53)	< 0.001
single solid line	1.45 (1.22 to 1.71)	< 0.001	1.54 (1.31 to 1.82)	< 0.001
double solid line	2.18 (1.29 to 3.68)	0.004	2.21 (1.31 to 3.75)	0.003
Road material				
sand/clay	reference		reference	
asphalt	1.89 (1.34 to 2.66)	< 0.001	1.95 (1.39 to 2.73)	< 0.001
Land use				
residential	reference		reference	
nonresidential	2.12 (1.90 to 2.37)	< 0.001	2.15 (1.93 to 2.40)	< 0.001
other uni-purpose areas	1.58 (1.39 to 1.79)	< 0.001	1.57 (1.38 to 1.78)	< 0.001
multi-purpose areas	1.24 (1.05 to 1.46)	0.012	1.25 (1.06 to 1.47)	0.007
Crash mechanism				
multiple-vehicle crash	reference		reference	
single-vehicle crash	1.13 (2.45 to 4.10)	0.051	1.20 (1.08 to 1.33)	< 0.001
involving vulnerable road users crash	1.70 (1.25 to 1.99)	< 0.001	1.70 (1.50 to 1.92)	0.001
View obstacles				
no	reference			
yes	0.98 (0.79 to 1.23)	0.901		
Crash position in the riding lane				
yes	reference			
no	1.08 (0.95 to 1.23)	0.224		
Dry road surface				
yes	reference			
No	0.99 (0.73 to 1.37)	0.978		
Curved geometric design				
No	reference			
yes	1.11 (0.99 to 1.25)	0.081		
Vehicle factor				
no	reference			
yes	0.69 (0.47 to 1.03)	0.068		
Human factor				
no	reference		reference	
yes	1.11 (1.02 to 1.21)	0.017	1.13 (1.03 to 1.23)	0.007
First cause				
irresponsibility	reference		reference	
need for more training	0.83 (0.75 to 0.91)	< 0.001	0.83 (0.75 to 0.92)	< 0.001
need for more training & irresponsibility	1.46 (1.30 to 1.63)	< 0.001	1.46 (1.30 to 1.64)	< 0.001
failure of organs	1.26 (0.59 to 2.68)	0.544	1.28 (0.61 to 2.68)	0.509
multiple factors	2.82 (2.04 to 3.89)	< 0.001	2.81 (2.04 to 3.87)	< 0.001
Prior cause				
hasty driving & lack of attention to driving	reference		reference	
lack of attention to driving	0.93 (0.85 to 1.01)	0.096	0.93 (0.85 to 1.01)	0.095
hasty driving	1.02 (0.89 to 1.17)	0.774	1.03 (0.89 to 1.18)	0.704
lacked skill	1.17 (0.98 to 1.40)	0.089	1.16 (0.97 to 1.39)	0.097
other	1.50 (1.29 to 1.75)	< 0.001	1.48 (1.27 to 1.72)	< 0.001
Direct cause				
regulation	reference		reference	
delay in sighting	1.34 (1.19 to 1.50)	< 0.001	1.35 (1.20 to 1.51)	< 0.001
overspending	1.06 (0.91 to 1.22)	0.468	1.05 (0.91 to 1.21)	0.496
Escaping crash in wrong way or multiple factor	1.07 (0.76 to 1.50)	0.704	1.06 (0.75 to 1.49)	0.738
Collision type				
side-swipe	reference		reference	
head-on	3.34 (2.85 to 3.91)	< 0.001	3.35 (2.85 to 3.93)	< 0.001
rear-end	1.06 (0.91 to 1.24)	0.454	1.06 (0.91 to 1.25)	0.450
T-bone	1.16 (0.99 to 1.36)	0.073	1.16 (0.99 to 1.36)	0.076
fixed-object	2.30 (1.81 to 2.91)	< 0.001	2.36 (1.87 to 2.99)	< 0.001
Crash province				
Tehran	reference		reference	
Isfahan	1.48 (1.30 to 1.68)	< 0.001	1.47 (1.30 to 1.67)	< 0.001
Fars	1.96 (1.70 to 2.26)	< 0.001	1.95 (1.69 to 2.24)	< 0.001
Razavi Khorasan	1.13 (0.99 to 1.30)	0.075	1.12 (0.97 to 1.28)	0.112
khuzestan	1.83 (1.60 to 2.09)	< 0.001	1.83 (1.60 to 2.09)	< 0.001
East Azerbaijan	0.93 (0.77 to 1.13)	0.459	0.93 (0.77 to 1.12)	0.451
Commuting area				
urban	reference		reference	
suburban	3.21 (2.78 to 3.70)	< 0.001	3.18 (2.76 to 3.67)	< 0.001
rural road	3.31 (2.69 to 4.08)	< 0.001	3.26 (2.65 to 4.01)	< 0.001
exclusive urban area	1.75 (0.82 to 3.76)	0.149	1.71 (0.80 to 3.67)	0.169
exclusive suburban area	3.08 (1.96 to 4.84)	< 0.001	3.04 (1.94 to 4.78)	< 0.001
Road type				
main street	reference		reference	
freeway	1.36 (1.09 to 1.68)	0.006	1.35 (1.09 to 1.68)	0.006
expressway	1.83 (1.58 to 2.11)	< 0.001	1.84 (1.59 to 2.13)	< 0.001
side street	1.22 (0.99 to 1.52)	0.066	1.22 (0.98 to 1.51)	0.076
main road	1.82 (1.59 to 2.09)	< 0.001	1.83 (1.59 to 2.10)	< 0.001
side road	1.39 (1.17 to 1.64)	< 0.001	1.39 (1.17 to 1.65)	< 0.001
rural road	1.38 (1.12 to 1.69)	0.003	1.34 (1.09 to 1.65)	< 0.001
alley	1.17 (0.89 to 1.65)	0.062	1.18 (0.88 to 1.64)	0.067
Road shoulder				
paved with asphalt	reference		reference	
paved with soil	1.37 (1.22 to 1.52)	< 0.001	1.38 (1.24 to 1.54)	< 0.001
unpaved	1.81 (1.61 to 2.05)	< 0.001	1.84 (1.63 to 2.07)	< 0.001
Road design				
one-way road	reference		reference	
separated two-way road	1.34 (1.20 to 1.49)	< 0.001	1.34 (1.20 to 1.50)	< 0.001
unseparated two-way road	1.42 (1.27 to 1.58)	< 0.001	1.40 (1.26 to 1.56)	< 0.001
Road defect				
no	reference		reference	
signs defects	1.70 (1.42 to 2.04)	< 0.001	1.72 (1.43 to 2.06)	< 0.001
geometric defects	1.23 (1.00 to 1.50)	0.045	1.26 (1.03 to 1.53)	0.023
pavement/ lightning defects	1.42 (1.05 to 1.92)	0.024	1.43 (1.06 to 1.94)	0.020
multiple defects	1.99 (1.66 to 2.39)	< 0.001	2.00 (1.67 to 2.39)	< 0.001
Road repairing				
no	reference			
yes	0.87 (0.63 to 1.19)	0.377		
Vehicle type				
light	reference		reference	
heavy	1.42 (1.27 to 1.58)	< 0.001	1.40 (1.26 to 1.56)	< 0.001
tricycle/ bicycle	0.43 (0.36 to 0.52)	< 0.001	0.42 (0.35 to 0.50)	< 0.001
Vehicle safety equipment				
yes				
no	0.91 (0.81 to 1.02)	0.097		
Vehicle color				
low risk	reference		reference	
high risk	1.26 (1.17 to 1.36)	< 0.001	1.26 (1.17 to 1.35)	< 0.001
Vehicle life				
10 to 14 yrs	reference		reference	
less than 5yrs	0.89 (0.80 to 0.99)	0.025	0.90 (0.81 to 1.00)	0.054
5 to 9 yrs	0.72 (0.65 to 0.78)	< 0.001	0.72 (0.66 to 0.79)	< 0.001
15yrs and more	1.46 (1.28 to 1.68)	< 0.001	1.46 (1.27 to 1.67)	< 0.001
Vehicle plaque description				
personal regional	reference		reference	
other	2.73 (2.41 to 3.08)	< 0.001	2.73 (2.42 to 3.09)	< 0.001
Vehicle moving direction				
cardinal direct	reference			
ordinal direction	1.18 (0.85 to 1.63)	0.337		
Vehicle maneuver				
turn	reference		reference	
forward	1.24 (1.00 to 1.53)	0.050	1.21 (0.98 to 1.49)	0.080
overtake	2.17 (1.25 to 3.76)	0.006	2.22 (1.28 to 3.84)	0.004
backward	1.95 (1.29 to 2.94)	0.002	1.89 (1.25 to 2.85)	0.003
stop on the road	3.21 (2.18 to 4.72)	< 0.001	3.08 (2.11 to 4.51)	< 0.001
other	3.90 (2.75 to 5.54)	< 0.001	3.84 (2.72 to 5.44)	< 0.001
Driver fault status				
not at fault	reference			
at fault	1.05 (0.96 to 1.15)	0.328		
Driver gender				
female	reference			
male	1.02 (0.84 to 1.23)	0.868		
Driver education				
academic	reference		reference	
illiterate	1.30 (0.97 to 1.73)	0.078	1.28 (0.96 to 1.71)	0.093
primary	1.26 (1.00 to 1.58)	0.048	1.24 (0.99 to 1.56)	0.063
nonacademic	1.60 (1.64 to 1.90)	< 0.001	1.58 (1.33 to 1.88)	< 0.001
Driver job				
jobs with high income	reference		reference	
jobs with middle income	1.49 (1.22 to 1.83)	< 0.001	1.49 (1.22 to 1.81)	< 0.001
jobs with low income	2.48 (1.94 to 3.18)	< 0.001	2.48 (1.95 to 3.15)	< 0.001
Driver age				
adult	reference		reference	
child	1.07 (0.80 to 1.43)	0.658	1.08 (0.81 to 1.45)	0.598
elderly	1.50 (1.27 to 1.77)	< 0.001	1.50 (1.26 to 1.77)	< 0.001
Driver license				
motorcycle	reference		reference	
class A	2.37 (1.52 to 3.71)	< 0.001	2.40 (1.54 to 3.75)	< 0.001
class B	1.88 (1.22 to 2.91)	0.004	1.90 (1.23 to 2.94)	0.004
class C	2.97 (2.25 to 4.62)	< 0.001	3.03 (2.28 to 4.71)	< 0.001
no license	3.91 (2.50 to 6.12)	< 0.001	3.93 (2.51 to 6.15)	< 0.001
Driver seat belt				
used	reference		reference	
not used	1.55 (1.45 to 1.67)	< 0.001	1.55 (1.44 to 1.67)	< 0.001
Driver judiciary cause				
carelessness	reference		reference	
other	1.64 (1.41 to 1.91)	< 0.001	1.67 (1.44 to 1.94)	< 0.001
Driver misconduct				
spiral movement	reference		reference	
over speeding	1.31 (1.35 to 1.52)	< 0.001	1.29 (1.33 to 1.50)	< 0.001
other	2.35 (2.47 to 2.84)	< 0.001	2.51 (2.39 to 2.88)	< 0.001

*OR* Odds ratio, *CI* Confidence interval

#### Passenger and pedestrian involved in a crash

In Table 3 review, crashes with passengers were 4.95 times, and crashes with pedestrians were 2.60 times more prone to fatal crashes. The presence of passengers may reduce attention to the driving task and exert direct or indirect psychological pressure to drive on less safe roads. In the same vein, it can be assumed that the presence of a passenger may lead to increased stress and, thus, reduced driving performance [20]. In addition, pedestrians are highly likely to be more vulnerable compared to other road users because they are less protected than the occupants of closed vehicles. The relatively high vulnerability of pedestrians to traffic accidents in metropolitan areas is consistent with the results of international research [21].

#### Crash-level variables

The odds ratios of day factor (limited to the weekend and weekday categories), zone type, view obstacles, crash position, road surface, road geometric design, vehicle factor, and road repairing status were not significant in resulting in fatal crashes (all P > 0.05).

Night time followed by twilight/ dawn time, was riskier than daytime (the odds of fatal crashes being at least 1.48 times greater). This may be held supported by the fact that there is high traffic volume during the daytime, which prevents drivers from driving at high speeds. On the other hand, driving during the day provides a better visual perception and more time to distinguish barriers and react. These conditions make drivers more cautious and better prepared to take necessary measures to reduce the risk of a severe crash.

Compared to clear/cloudy weather, the odds of fatal crashes increased by 1.32 times during rainy weather. Meanwhile, snowy weather was 65% less prone to a fatal crash. Foggy/stormy/dusty and clear/cloudy weather conditions were equally likely to lead to fatal crashes. Although the number of road collisions on snowy and rainy days is inevitably higher than on clear and cloudy days, and driving on these days is more dangerous due to limited visibility and tire adhesion, drivers drive more carefully and at lower speeds. In addition, most people avoid unnecessary travel or postpone it to another time. For these reasons, the available documents suggest that less severe traffic accidents (property damage or injury) increase on snowy days, and more severe ones (fatality) increase on these days.

As in previous research [22], the results indicate that roads without specific traffic control are severe road features with high odds of resulting in fatality if a collision occurs. The absence of intersection control has led to a higher possibility of fatal crashes (1.40 times more). Intersection control can force drivers to comply with traffic control. As a salient example, after detecting a vehicle proceeding inside the intersection with not yielding the right of way, the officer can stop and issue a citation for the noncompliant driver. Such targeted enforcements increase legitimacy among offenders and others who observe or hear about these activities.

The line marking showed a significant effect. Broken lines were 1.36 times more likely to induce fatal crashes than crash locations with no line marks. Subsequently, single and double solid lines were even more critical than broken ones: they were 1.54 and 2.21 times more likely to lead to fatal crashes, respectively. This could be related to the fact that double solid lines mark the boundaries of each way on two-way roads where the risk of a head-on collision and, consequently, death is much higher on these roads.

Regarding road material, asphalt roads were ~ 2 times more likely to result in fatal crashes when compared to sand/clay roads. Drivers are less cautious and alert to their performance, especially regarding speed control when driving on asphalt roads, since they possess more good situations than other road types. This finding is in line with existing literature [23].

Consistent with existing studies [24, 25], findings from the present study indicate that crashes happening in non-residential areas exacerbate the crash outcome more than in other regions (being 2.15 times more fatal). While driving in non-residential areas, drivers are more likely to engage in risky driving behaviors since they usually do not perceive a critical situation in non-residential areas.

Crash severity analysis based on collision mechanisms revealed that involving vulnerable road users was associated with more severe crashes. Since they are directly exposed to impact, they succumb to death and increase the fatality chance.

The results also revealed that it was 13% more likely to die in the presence of human factors in the causation of a road traffic crash. Similar studies showed that human factors (namely: hasty driving, ignoring traffic regulations, fatigue, drowsiness, etc.) were the sole cause of many accidents [26, 27].

When dealing with judiciary causation factors:

In terms of the first cause, except needing more training, simultaneity of needing more training combined with irresponsibility and other multiple factors played a critical role in increasing the odds of a fatal crash as compared to irresponsibility solely. Failure of organs was almost the same as irresponsibility resulting in deadly crashes. Policymakers have applied numerous measures to alleviate the severity of traffic crashes, the very epitome of which could be speed cameras and police surveillance. Having said that, pedagogical approaches planned for drivers are another way to cultivate more safe drivers by letting them know about traffic safety and improving their driving skills.

Considering prior causes showed that other factors, namely, fatigue and drowsiness, lack of skill in diagnosing traffic situations, slippery or tarred road surfaces, etc., have increased the odds of dying in a road traffic crash by 50%. To elaborate, it is believed that, after drunken driving, drowsiness is the most prominent cause of vehicle accidents. However, many experts believe this is only a conservative estimate, and the actual contribution of fatigue and sleepiness to vehicle accidents may be higher [28]. Sleepiness is a component of sleep in the circadian rhythm of sleep and wakefulness. Drowsiness leads to driving automobile accidents because it can impair performance and ultimately lead to the inability to deal with falling asleep behind the wheel. Although sleeping is the most effective way to reduce drowsiness, sometimes it is unavoidable, particularly for professional drivers, to continue driving for some reasons like shift work [29]. Accidents caused by fatigue and drowsiness are often severe and have a significant financial burden and catastrophic personal consequences. Therefore, researchers have proposed effective solutions to reduce this problem, including educational activities, behavior changes, and environmental changes [28].

Talking about direct causes, it can be inferred that delay in sighting was the only cause that significantly increased (1.35 times) the odds of a fatal crash compared to irregulation. Delayed vision can be due to drivers’ health disorders, particularly adult attention-deficit/hyperactivity disorder (ADHD) or their visual impairment. Symptoms of ADHD namely lack of focus, hyperfocus and disorganization have been proven to be related with crash severity [30]. Studies have proposed that impulsiveness and visual inattentiveness are the main contributions to the severity of car accidents in patients with ADHD. In addition, therapies that mitigate ADHD symptoms translate into more safe driving behavior and accordingly decreased rates of serious crash severity [31]. Supplementary to this, it has been proved that drivers with poor visual acuity are more prone to road traffic crashes [32]. Estimated number of crashes contributed to visual field defect has been reported to be 36% higher. With regard to protanopic color vision defect, it is not allowed for people with theses defect to obtain a commercial license since they cannot diagnosis red traffic lights [33]. In the light of above-mentioned descriptions, mental stability and visual functioning of drivers seems to have inevitable results in road traffic crashes and would be fundamental issue that needs to be taken under more consideration.

Compared to a side-swipe collision, within a head-on collision followed by a fixed object collision, the odds of fatal crashes significantly increased by 3.35 times and 2.36 times, respectively. Meanwhile, rear-end and T-bone collisions were almost the same as side-swipe collisions regarding crash severity. Consequences of head-on collision could hurt the driver directly in numerous ways, exacerbating the crash outcome and even leading to fatal crashes. Head-on collisions are the type of crashes with the utmost severity and often lead to injuries and fatalities [34].

Compared to Tehran, the capital city of Iran, Fras, Khuzestan and then Isfahan were accounted for the most risky provinces in Iran where about 62% of all fatal crashes occurred in these provinces. Isfahan, Iran’s top tourist destination, provides a classic tourist stop on a travel itinerary from northern cities of Iran to the southern tourist city of Shiraz in Fars province. In addition, these two provinces are attractive tourist destinations for outbound visitors. Accordingly, these two provinces, with high traffic volume and different driving characteristics (risky driving behaviors, drowsy driving, high speed, etc.), exhibit higher rates concerning crash severity and even fatality. On the other hand, in Khuzestan, as a Border city, drivers tend to use foreign cars, leading them to drive more speedily. Other studies show that high speed is crucial in causing severe crashes. Furthermore, a greater fatality rate in these three provinces could be attributed to the following issues: (1) emergency medical services performance. In this regard, the number of at-scene, on-transfer, and in-hospital deaths had better be considered, (2) unsafe roads, (3) higher rates of heavy vehicles, and pedestrian and pedestrian and motorcycle crashes.

The results also showed that commuting areas contributed to crash severity. Going into detail revealed that suburban regions were at least three times more likely to result in fatal crashes when compared to urban areas. It has been reported that crashes occurring on rural roads produce lucid trend patterns toward more severe and even fatal crashes. It is believed that the features of the rural highway, such as rural drivers’ typical behaviors (less likely to wear a seat belt, more incredible driving speeds or stop at stop-sign intersections, etc.) and their characteristics (more older drivers or the adversity of reaching in time medical assistance in the time of crash) are leading factors to more frequent fatal crashes on rural roads [35, 36].

Compared to the main street, a crash was more likely to involve fatality in an expressway, main road, side road, freeway, and rural road, respectively. These road types are commercial and in suburban areas. Alongside line marking in the aforementioned areas is a double solid line. And, as has already been shown in the previous results of this study, the crash outcome is more severe in suburban areas and roads with double solid lines.

In addition, considering shoulder condition and design of the roads, roads with unpaved shoulders and separated two-way roads contributed to higher risk. Road shoulder provides a necessary stopping lane and serves recovery for errant vehicles beforehand a potential crash occurs. Its omission could hence lead to more severe collisions. Furthermore, unseparated two-way roads, like roads with double solid lines, are more likely to have head-on collisions that are more prone to fatality.

In completing the crash-level variables, it should be mentioned that in addition to the factors discussed above, coincidences of multiple road defects, such as signs, geometric defects, etc., implied a higher risk of fatal crashes (Table 3). Road defects are those where a road design element transfers ambiguous information to drivers, resulting in driver error, or where a change in the road could have reduced the likelihood of a road accident. It has been previously reported that road environments that encourage risky driver behavior (e.g., by inspiring high traffic speeds) or fail to consider safety in all conditions (e.g., at night or in adverse weather conditions) increase the probability of a road accident and its severity indirectly. Hence, a road that is designed and regularly maintained according to operational and functional requirements is critical in influencing drivers’ perceptions and resulting in safer roads for all users [37]. It has been shown that the road environment element is in poor condition in developing countries due to worse road design and maintenance. In addition, defects of various traffic combinations requiring different infrastructure needs are commonly not observed on roads such as high-speed vehicles, heavy marketable traffic, bicyclists, pedestrians, and motorcycle users [38]. However, the growing number of motorized vehicles in developing countries is outstripping the capacity of current transportation infrastructure, leading to increased accident rates and severity levels.

### Vehicle-level variables

The following rows in Table 3 provide results regarding vehicle factors. It can be observed that vehicle safety equipment and moving direction were not associated with a fatal crash happening. On the other side, the categories highly related to crashes involving fatality were: heavy vehicle type, vehicles of risky colors with life of fifteen years and more, vehicles with no personal regional plaques, and vehicles with maneuvers such as stopping outside of the road, sudden starting, sudden stopping, and spiral movement.

Although heavy vehicle crashes are less frequent, these crashes are more severe to such an extent that approximately 18 percent of all fatal crashes in 2019 involved heavy vehicles [39]. Intense exposure is the leading cause of severe injury or even death in heavy vehicle accidents. It is also noteworthy that these accidents often lead to the death of the users of the other vehicle [40].

The findings about silver color for cars, in particular, clearly contrast with results of a case–control study which concluded that silver vehicles were approximately 50% less prone to serious crashes compared to colored cars. The results of this study are biased due to not considering several critical confounding factors such as vehicle type and personality traits of drivers. It is stated that commercial vehicles that are more likely to severe crashes are predominantly white. Secondly, there might be a relationship between driving behavior and color choice. For instance, more careful drivers may prefer silver color. In contrast, in a paired case–control study, the authors concluded that vehicles with light colors were associated with less dangerous collisions. Although this study tried to account for particular driver and vehicle features and consider many confounders, it failed to consider unmeasurable or unmeasured confounders. It has also been stated that white color, black, blue, grey, green, silver, and red were associated with more serious crashes. This association was even more vital during daylight than in the dark or twilight times [41].

Consistency results exist about vehicle age. The studies assessing the impact of vehicle age on car collisions have found that older vehicles are more prone to be included in severe crashes. It has been proved that older vehicles, as compared to new ones, are more likely to develop defects in terms of safety, like brake failure and tire. On the other hand, older vehicles are less likely to have safety features. Safety equipment and its defects cause a crash and may increase its intensity [42].

The difference between personal and commercial vehicles could be attributed to the fact that commercial vehicles are heavy, and their drivers suffer from sleepiness and fatigue more than private vehicles. On the other hand, commercial cars are usually on highways, expressways, and main roads, which are more critical for intense crashes. In addition, unusual maneuvers such as stopping outside the lane, sudden starting, sudden stopping, and spiral movement are categorized as risky driving behaviors, strongly linked with crash severity.

### Driver-level variables

Table 3 also presents results about driver-level factors. Driver fault status and gender were not significant in predicting a fatal crash. The categories with the highest odds ratio of deadly crashes were: divers with non-academic education and middle-income status, driver old age, no driving license, not using the seat belt, driver unconscious or lack of driving skills or violation of the law, and driver misconduct other than spiral movement or over speeding.

Driver education and income, as well as socioeconomic status (SES), play a crucial role in the breakthrough of traffic safety. Cognitive perception, which constructs the way of interpreting and understanding different situations and whether being obedient to rules, is closely related to SES. A driver with a high SES level would hardly ever be under too extreme fear and courage sense and perform more reasonably in a critical situation [43]. Beyond behavioral factors, vehicle-related and contextual features can be attributed to the exacerbated risk among individuals with low SES. These people usually suffer from monetary crises to such an extent that they can barely cope with their day-to-day expenses. An inevitable result of being riddled with such unaffordability would possess a vehicle with no advanced safety equipment and a lower crash-test rating. On the other hand, are properties might also have relevance, as there is a striking difference in accessibility to hospital trauma centers between common and high-property areas. Limited access to trauma centers and specialists may increase crash severity and the following mortality rate [44].

It is undoubtedly true that, nowadays, increased traffic crashes among the elderly have become pervasive among a thorough of nations all around the globe [45]. Suffering from musculoskeletal disorders and slowed physical activities, older people experience more severe crash outcomes than the middle-aged group in the case of traffic collisions. Furthermore, chronic diseases, the very epitome of osteoporosis, increase the rate of bone fracture and consequently extend the hospitalization period and mortality rate in this generation. This predicament imposes an undue financial burden on the healthcare system of each society by increasing medical costs. So, to stave off this deleterious condition and enhance traffic safety, particular policies had better be the matter of greater emphasis for this age group.

Consistent driving license results prove that unlicensed operators are more likely to be involved in a severe crash and engage in illegal behaviors such as red light running, speeding, drunken driving, and not using a seat belt. Also, these groups are more prone to be at fault than licensed drivers. Since unlicensed drivers are on the rise, measures such as increasing petrol enforcement, expanding the applied penalties, and promoting public knowledge about the dangers of driving without a license and vehicle impoundment need to be taken for this population [46].

Turning to seat belt use, it is evident that seat belt use can considerably decrease non-fatal and fatal injuries both in front and rear seat occupants. In a study, authors found that people in metropolitan and urban areas are likelier than those in rural areas to use seat belts. In addition, gross provincial product, educational level, and legalization were declared to be related to the use of seat belts [47].

### Key results and insights

**Fig. 2:**
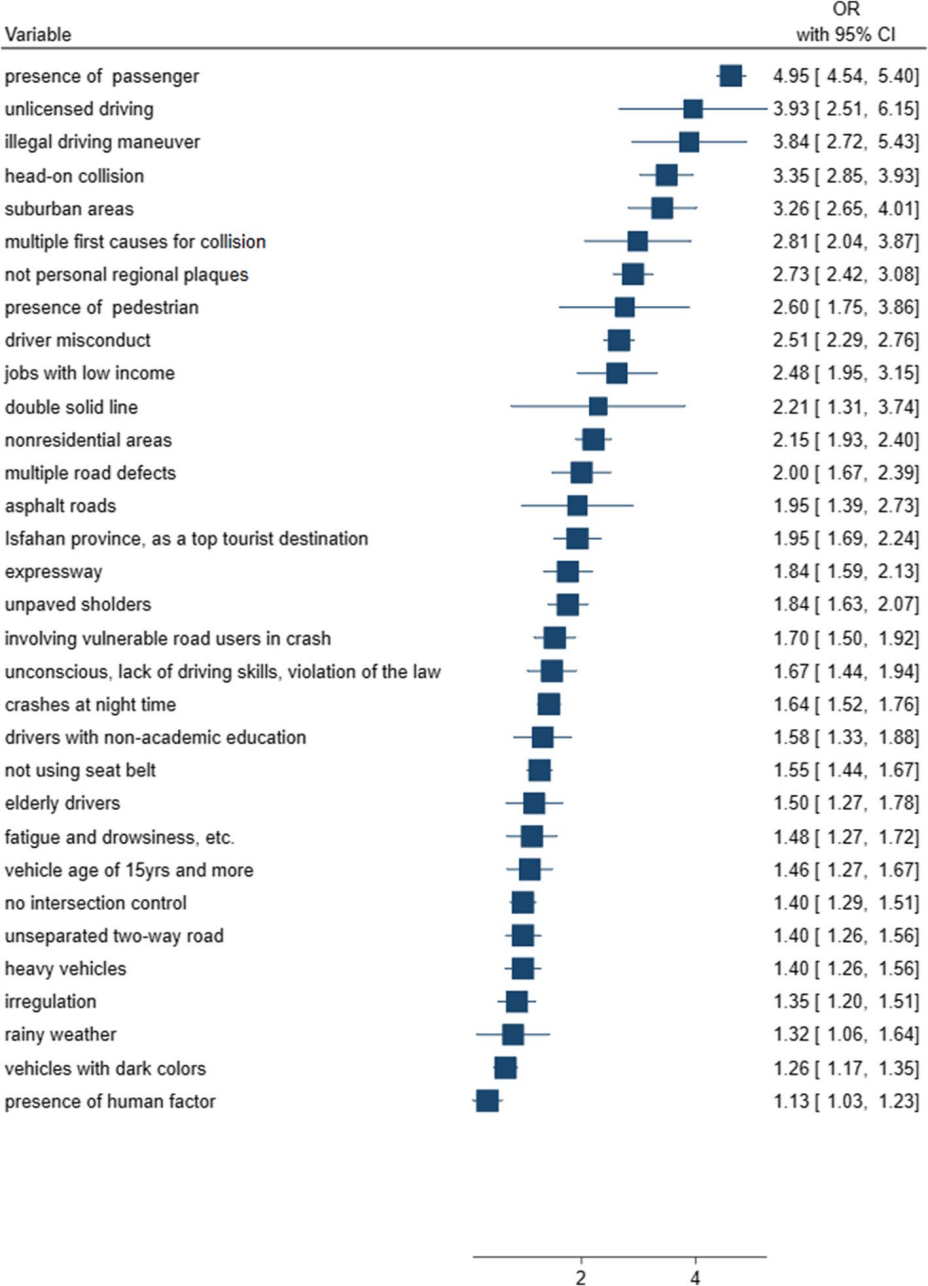
Explored risk factors and odds ratios in predicting road traffic fatalities in Iran, 2015–2016

Table 4 summarizes the significant factors, their level, and the safest categories contributing to fatal crashes based on the multiple logistic regression model. For better figurative presentation, explored risk factors and corresponding odds ratios are illustrated in Fig. 2.

**Table 4:** Summary of main factors increasing fatal crashes

Factor	Type of factor	Most dangerous category	OR (95% CI)
Passenger include	Passenger level	presence of passenger	4.95 (4.54 to 5.40)
Pedestrian include	Pedestrian level	presence of pedestrian	2.60 (1.75 to 3.85)
Lightning status	Crash level	night	1.64 (1.52 to 1.76)
Weather	Crash level	rainy	1.32 (1.06 to 1.64)
Intersection control	Crash level	no intersection control	1.40 (1.29 to 1.51)
Line marking	Crash level	double solid line	2.21 (1.31 to 3.75)
Road material	Crash level	asphalt	1.95 (1.39 to 2.73)
Land use	Crash level	nonresidential	2.15 (1.93 to 2.40)
Crash mechanism	Crash level	involving vulnerable road users crash	1.70 (1.50 to 1.92)
Human factor	Crash level	presence of human factor	1.13 (1.03 to 1.23)
First cause	Crash level	multiple factors	2.81 (2.04 to 3.87)
Prior cause	Crash level	other factors (e.g., fatigue and drowsiness, lack of skill in diagnosing traffic situation, slippery or tarred road surface, etc.)	1.48 (1.27 to 1.72)
Direct cause	Crash level	irregulation	1.35 (1.20 to 1.51)
Collision type	Crash level	head-on collision	3.35 (2.85 to 3.93)
Crash province	Crash level	Isfahan	1.95 (1.69 to 2.24)
Commuting area	Crash level	suburban	3.26 (2.65 to 4.01)
Road type	Crash level	expressway	1.84 (1.59 to 2.13)
Road shoulder	Crash level	unpaved	1.84 (1.63 to 2.07)
Road design	Crash level	unseparated two-way road	1.40 (1.26 to 1.56)
Road defect	Crash level	multiple defects	2.00 (1.67 to 2.39)
Vehicle type	Vehicle level	heavy vehicles	1.40 (1.26 to 1.56)
Vehicle color	Vehicle level	dark colors	1.26 (1.17 to 1.35)
Vehicle life	Vehicle level	15yrs and more	1.46 (1.27 to 1.67)
Vehicle plaque description	Vehicle level	not personal regional plaques	2.73 (2.42 to 3.09)
Vehicle maneuver	Vehicle level	maneuver such as stopping outside of the road, sudden starting, sudden stopping, overtaking, spiral movement	3.84 (2.72 to 5.44)
Driver education	Driver level	non-academic	1.58 (1.33 to 1.88)
Driver job	Driver level	jobs with low income	2.48 (1.95 to 3.15)
Driver age	Driver level	elderly	1.50 (1.26 to 1.77)
Driver license	Driver level	no license	3.93 (2.51 to 6.15)
Driver seat belt	Driver level	not used	1.55 (1.44 to 1.67)
Driver judiciary cause	Driver level	causes such as unconscious, lack of driving skills, violation of the law	1.67 (1.44 to 1.94)
Driver misconduct	Driver level	misconducts such as failure to yield right-of-way, failure to yield right-of-way, failure to distance control while overtaking, running red light, passing the prohibited place, illegal overtaking, turning left or right in the prohibited place, turning in the prohibited place, drunken driving, lack of safety equipment for the season, not turning on the lights from sunset to sunrise, not using glasses while driving, defective vehicle lighting system at night, demonstrative movement, crossing the sidewalk	2.51 (2.39 to 2.88)

### Strength and limitation

All registry system variables are presented in Table 1, whether they were used as an explanatory variables or not. Since most categories are based on international classifications, they can be considered referral documents for developing traffic crash registry systems in other countries. In this study, we evaluated unknown values missing and replaced them with missing data management strategies. Although added values such as shoulder width, road width, and road length were not included in the study due to a significant error in recording information, variables such as speed limit, road type (one-way, two-way, etc.) and road type (expressway, freeway, etc.) were an excellent representative of these added values and did not affect the results significantly.

Focusing on the data between 2015 and 2016 and a restriction to access data from 2016 to 2021, which would enlarge and improve this research, can be considered the main limitation of this study.

### Recommendations

The crash location’s longitude and latitude had many missing values and could not be taken into consideration for related analysis and detecting more gangrenous segments. Considering some limitations, such as defining upper and lower limits in recording the aforementioned quantitative variables, is suggested in designing, developing, or editing traffic crash registry systems. In addition, the registry system had better be provided via advanced features such as automatic fulfillment of road length and width or shoulder width by selecting road name and type. If so, researchers could use this critical information in the more complicated and specialized analysis. Furthermore, it is worse to notice that, although comparing factors in overall analysis is sound, subgroup analyses sometimes provide better specific information, such as modeling of factors affecting road traffic injuries in expressways’ head-on collisions that may lead to more specified decisions for giving areas. So subgroup analyses regarding all significant identified aspects are suggested for further investigation. Since machine learning (ML) methods can be used for prediction peruses and overcome the limitations associated with traditional statistical models, applying ML approaches in recommended subgroup analyses would be of the utmost practicality. Beyond this, more specific analyses about passenger and pedestrian fatalities are also suggested.

## Conclusions

In this study, the effect of seventy-one different features from the aspect of crash scene, vehicle, driver, passenger, and a pedestrian was assessed to find their connection with crash outcome in 384,614 collision crashes in the six provinces with the largest population of a developing country; Iran from 2015 to 2016. There was 32 variable to be significantly correlated with fatal crash occurrence. Although road traffic injuries contribute to a global problem, it is more challenging in low- and middle-income counties to such an extent that more than 90% of world fatalities due to collision crashes occur in these counties [48]. Information regarding road collisions was available in the separate crash scene, vehicle, driver, passenger, and pedestrian databases, which are now combined. This provides an opportunity to compare all the factors in the overall analysis that may lead to comprehensive decisions. According to the multiple binary logistic regression model, many variables included in the analysis played a significant role in crash severity. The top factors with an odds ratio of at least two which contribute to fatal crashes are the presence of a passenger, unlicensed driving, illegal driving maneuver, head-on collision, crashes in suburban areas, the occurrence of multiple causes for collision, vehicles with not personal-regional plaques, presence of pedestrians, drivers with low-income jobs, driver misconduct, roads with double solid lines, non-residential areas, multiple road defects. Looking more closely at the most significant factors reveals that they are primarily from driving behavior (presence of passenger, unlicensed driving, illegal driving maneuver, occurrence of multiple causes for collision, vehicles with not personal-regional plaques, presence of pedestrians, drivers with low-income jobs, driver misconduct, head-on collision), infrastructure design (roads with double solid lines and multiple road defects), and geometric road factors (crashes in suburban areas, non-residential areas). The quantitative values of the impact of the significant features obtained in this study can provide unique guides or recommendations for road managers and policymakers for prioritizing measures to prevent fatal crashes. We believe that the result of this study can be considered for proper and well-designed measures to prevent fatal crashes.

## Data Availability

The dataset created and support the findings of the current study are not accessible by the general public since not requesting consent during the study protocol submission and from participants. However, they are available from the corresponding author upon reasonable request.
