# Correction to: modeling road traffic fatalities in Iran’s six most populous provinces, 2015–2016

**DOI:** 10.1186/s12889-023-16323-1

**Published:** 2023-10-09

**Authors:** Fatemeh Jahanjoo, Homayoun Sadeghi-Bazargani, Mohammad Asghari-Jafarabadi

**Affiliations:** 1 Road Traffic Injury Research Center, Tabriz University of Medical Sciences, Tabriz, East Azerbaijan, 5167846311 Islamic Republic of Iran; 2 Injury Epidemiology and Prevention Research Group, Turku Brain Injury Center, Turku University Hospital and the University of Turku, Turku, Finland; 3 Cabrini Health, Melbourne, VIC 3144 Australia; 4 School of Public Health and Preventative Medicine, Faculty of Medicine, Nursing and Health Sciences, Monash University, Melbourne, VIC 3800 Australia

The original publication of this article contained an incorrect funding section. The correct and incorrect information is listed in this correction article, the original article has been updated

Incorrect

Not applicable.

Correct

This study was based on data from Fatemeh Jahanjoo’s Ph.D. thesis, which was financially supported by the Research Deputy of the Tabriz University of Medical Sciences (TUOMS) under Grant No. 64041 and approved by the Institutional Review Board of TUOMS with ethics code: IR.TBZMED.REC.1398.1244.

